# Season-matched comparison of pediatric respiratory pathogen detection patterns before and after the lifting of COVID-19 restrictions in Guangzhou

**DOI:** 10.3389/fped.2026.1755382

**Published:** 2026-07-03

**Authors:** Liang Hua, Wanli Liang, Wanling Li, Bing Zhu

**Affiliations:** Center Laboratory, Guangzhou Women and Children’s Medical Center, Guangzhou Medical University, Guangzhou, China

**Keywords:** COVID-19, epidemiology, multiplex probe amplification, pediatric respiratory infections, southern China

## Abstract

**Introduction:**

COVID-19-related non-pharmaceutical interventions and their subsequent relaxation may alter the epidemiology of pediatric respiratory pathogens. However, hospital-based data from subtropical southern China using a consistent multiplex PCR platform remain limited. This study described changes in pediatric respiratory pathogen detection patterns in Guangzhou, with emphasis on a season-matched comparison before and after the lifting of restrictions.

**Methodology:**

We retrospectively analyzed 2,213 children 0 to <16 years who presented with fever or respiratory symptoms at Guangzhou Women and Children's Medical Center. Nasopharyngeal swabs collected during the pre-lifting period (November 2021–October 2022) and post-lifting period (February–May 2023) were tested using the same specimen type, nucleic-acid extraction system, PCR instrument, and 12-pathogen multiplex PCR panel. To reduce seasonal bias, the primary pathogen comparison was restricted to February–May 2022 vs. February–May 2023. Categorical variables were compared using the Chi-square test or Fisher's exact test, as appropriate.

**Results:**

Overall, 843 of 2,213 children (38.1%) tested positive for at least one pathogen. Compared with February–May 2022, February–May 2023 showed higher detection rates of Influenza A (0.64% vs. 16.60%), RSV (5.72% vs. 14.67%), and HRV (1.06% vs. 5.79%), and lower detection rates of Influenza B (7.84% vs. 0.39%) and HMPV (10.59% vs. 0.39%). The post-lifting cohort included a higher proportion of infants (<1 year: 14.29% vs. 8.50%) and higher proportions of fever, wheezing, and lower respiratory tract diagnoses; these clinical comparisons should be interpreted cautiously because health-care-seeking behavior and admission thresholds may have changed after lifting restrictions.

**Conclusions:**

After the lifting of COVID-19 restrictions, pediatric respiratory pathogen detection in Guangzhou shifted toward increased Influenza A, RSV, and HRV activity in the season-matched comparison, with a younger patient composition and more lower-respiratory clinical diagnoses among tested children. These findings support continued multiplex surveillance and age-targeted prevention strategies, but causal attribution to the lifting of restrictions should be made cautiously because of residual confounding by seasonality, SARS-CoV-2 circulation, and health-care-seeking behavior.

## Introduction

The coronavirus disease 2019 (COVID-19) pandemic, which began in late 2019, prompted global efforts to mitigate its spread, including stringent lockdown measures, travel restrictions, and social distancing protocols (1–3). These measures drastically altered daily life and significantly impacted the transmission dynamics of not only the severe acute respiratory syndrome-coronavirus-2 (SARS-CoV-2) virus but also other respiratory pathogens (4). As children are particularly vulnerable to respiratory infections, the pandemic and its associated lockdowns raised concerns regarding the broader effects on pediatric health. While the focus has largely been on COVID-19, it is essential to examine how these interventions influenced the prevalence of other common respiratory viruses in children.

Respiratory infections are among the leading causes of morbidity and mortality in children, with a wide range of pathogens, such as Influenza A (FluA), respiratory syncytial virus (RSV), human rhinovirus (HRV), and human metapneumovirus (HMPV), causing varying degrees of illness (5–7). Before the pandemic, seasonal patterns of these infections were well-established, with certain viruses peaking at specific times of the year (7). However, the imposition of lockdown measures significantly disrupted these patterns, as social distancing, mask-wearing, and restricted mobility reduced the transmission of many viral pathogens. As a result, there was a marked decrease in the incidence of respiratory infections during the early years of the pandemic (4, 8), but with the easing of restrictions, questions arose about the potential resurgence of these infections and the subsequent changes in their transmission dynamics.

Although several recent studies have described post-pandemic changes in pediatric respiratory infections, the magnitude and direction of these changes vary by region, climate, local public-health policies, population immunity, and testing practices. Evidence from subtropical southern China remains important because respiratory virus seasonality differs from that in temperate regions, and the transition after the lifting of restrictions was accompanied by changes in hospital attendance and testing demand. Therefore, this study aimed to describe changes in the spectrum of respiratory pathogens among children in Guangzhou using a single-tube multiplex PCR assay capable of detecting 12 common respiratory pathogens. We focused on a season-matched comparison of February–May 2022 and February–May 2023 to reduce seasonal bias and to identify clinically relevant changes in pathogen spectrum, patient age composition, and hospital-based clinical presentation.

## Methodology

### Study design and participants

This retrospective observational study was conducted at Guangzhou Women and Children's Medical Center from November 2021 to May 2023. The full descriptive period was divided into a pre-lifting period (November 2021–October 2022) and a post-lifting period (February–May 2023). Because these periods differed substantially in duration and respiratory viruses have strong seasonality, the main comparison of pathogen-specific detection rates was restricted to the same calendar months, February–May 2022 vs. February–May 2023. The full one-year pre-lifting period was retained only to provide descriptive background and monthly trend visualization, not to infer incidence rates across unequal observation windows.

The study included 2,213 children aged 0 to <16 years who presented with respiratory symptoms (such as cough, sore throat, wheezing, or shortness of breath) or fever (>38.5℃) lasting ≥2 days and who underwent respiratory pathogen testing as part of routine clinical evaluation. Age groups were defined as <1 year, 1 to <3 years, 3 to <5 years, and 5 to <16 years for tabulated comparisons; the 5 to <16-year group was used because the number of detections in the older age strata was limited. Children with major chronic respiratory diseases or other conditions that could markedly alter pathogen susceptibility were excluded, including bronchopulmonary dysplasia, physician-diagnosed chronic asthma requiring long-term controller therapy, primary or secondary immunodeficiency, congenital heart disease with hemodynamic significance, chronic kidney or liver disease, malignancy, organ transplantation, or long-term systemic immunosuppressive therapy. Nasopharyngeal swabs were collected by trained health-care staff using standard aseptic techniques, immediately placed in transport medium, and processed within four hours of collection.

### Pathogen detection

Nucleic acids were extracted using a fully automated nucleic acid extractor (Daan Swift96, Guangzhou Daan Gene Co., Ltd., Guangzhou, China) following the manufacturer's instructions. Detection was performed using multiplex PCR on a SLAN 96P fluorescent quantitative PCR instrument (Shanghai Hongshi Medical Technology Co., Ltd., Shanghai, China). The same specimen type, extraction platform, PCR instrument, and multiplex PCR panel were used throughout the pre- and post-lifting periods. Twelve respiratory pathogens were tested using the BIOTRON Respiratory Panel 12 (Fluorescent PCR Melting Curve Method; Guangzhou Biotron Technology Co., Ltd., Guangzhou, China; Cat. No. 20243401400), including Influenza A virus (FluA), Influenza B virus (FluB), respiratory syncytial virus (RSV), human rhinovirus (HRV), human parainfluenza virus (HPIV), human adenovirus (HAdV), human metapneumovirus (HMPV), *Mycoplasma pneumoniae* (MP), *Chlamydia pneumoniae* (CP), human bocavirus (HBoV), human coronavirus (HCoV), and SARS-CoV-2. The results for HAdV, FluA, FluB, HPIV, RSV, and HCoV were not subtyped. A professional technician conducted testing and reported results in accordance with the manufacturer's instructions. Primer sequences are proprietary to the manufacturer and not publicly disclosed.

### Data collection and analysis

Data on demographic characteristics, including age, sex, and health-care setting (outpatient or inpatient), were collected from medical records. Clinical information, including fever, wheezing, upper respiratory tract diagnosis, lower respiratory tract diagnosis, and gastrointestinal disease, was also extracted. Upper and lower respiratory tract diagnoses were based on contemporaneous clinical diagnoses recorded in the medical record rather than adjudicated severity endpoints. All data were anonymized prior to analysis. The study was approved by the hospital's institutional review board, and informed consent was obtained from the guardians of all participants.

Statistical analysis was conducted using SPSS version 26.0 (IBM). Descriptive statistics, including frequencies and percentages, were used to summarize demographic and clinical characteristics. Because the full pre-lifting and post-lifting periods had unequal observation lengths, pathogen-specific comparisons were performed primarily between February–May 2022 and February–May 2023 to reduce seasonal bias. Categorical variables were compared using the Chi-square test or Fisher's exact test, as appropriate; Fisher's exact test was used when expected cell counts were small. Continuous age was summarized as median and interquartile range. Age distribution was analyzed using prespecified age groups (<1 year, 1 to <3 years, 3 to <5 years, and 5 to <16 years). The inpatient/outpatient and clinical-diagnosis comparisons were interpreted as changes in the tested hospital population rather than population incidence or disease severity, because health-care-seeking behavior, test accessibility, and admission thresholds may have changed after lifting restrictions. A two-sided *P*-value <0.05 was considered statistically significant.

## Results

**Figure 1:**
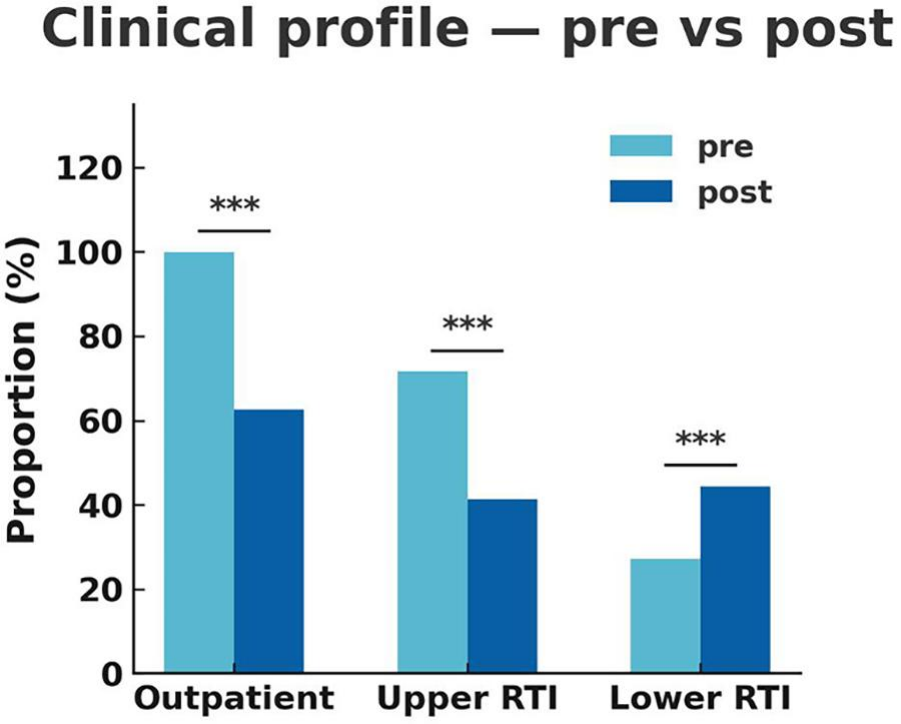
Comparison of clinical profiles before and after lifting of COVID-19 restrictions, including health-care setting and selected clinical diagnoses among tested children.

The demographic and clinical characteristics of the study population are summarized in Table 1. The post-lifting tested population differed from the pre-lifting population in health-care setting and clinical presentation: outpatients accounted for 99.90% before lifting and 62.55% after lifting, upper respiratory tract diagnoses decreased from 71.55% to 41.31%, and lower respiratory tract diagnoses increased from 27.18% to 44.40% (Figure 1). Fever and wheezing were also more frequent after lifting restrictions. These findings indicate a shift in the composition of children who underwent testing at the hospital; they should not be interpreted as population-level incidence or as definitive evidence of increased severity without adjustment for health-care-seeking behavior and admission thresholds. The proportion of infants <1 year increased from 8.50% to 14.29%. Overall, 843 of 2,213 children (38.09%) tested positive for at least one of the 12 pathogens. The most frequently detected pathogens were FluA, FluB, and HPIV, whereas MP, SARS-CoV-2, and CP were least frequently detected.

**Table 1:** Demographic and clinical characteristics of tested children before and after lifting COVID-19 restrictions.

Characteristic	Total (*n* = 2,213)	Before lifting (*n* = 1,954)	After lifting (*n* = 259)	*P*-value
Demographics				
Age, median (IQR), years	3 (0–11)	3 (0–11)	3 (0–10)	**0.011**
Age groups, *n* (%)				
<1 year, *n* (%)	203 (9.17)	166 (8.50)	37 (14.29)	**0.004**
1 to <3 years, *n* (%)	623 (28.15)	552 (28.25)	71 (27.41)	
3 to <5 years, *n* (%)	551 (24.90)	479 (24.51)	72 (27.80)	
5 to <16 years, *n* (%)	836 (37.78)	757 (38.74)	79 (30.51)	
Females, *n* (%)	979 (44.24)	863 (44.17)	116 (44.79)	0.902
Outpatients, *n* (%)	2,114 (95.53)	1,952 (99.90)	162 (62.55)	**<0.001**
Clinical symptoms and diagnoses				
Fever, *n* (%)	402 (18.17)	335 (17.14)	67 (25.87)	**0.001**
Wheezing, *n* (%)	97 (4.38)	50 (2.56)	47 (18.15)	**<0.001**
Upper respiratory tract infection, *n* (%)	1,505 (68.01)	1,398 (71.55)	107 (41.31)	**<0.001**
Lower respiratory tract infection, *n* (%)	646 (29.19)	531 (27.18)	115 (44.40)	**<0.001**
Gastrointestinal Disease, *n* (%)	91 (4.11)	73 (3.74)	18 (6.95)	**0.023**
Pathogen detection				
Pathogens detected, *n* (%)	843 (38.09)	719 (36.80)	124 (47.88)	**0.001**
Influenza A (FluA), *n* (%)	214 (9.67)	171 (8.75)	43 (16.60)	
Influenza B (FluB), *n* (%)	167 (7.55)	166 (8.50)	1 (0.39)	
Human parainfluenza virus (HPIV), *n* (%)	127 (5.74)	119 (6.09)	8 (3.09)	
Respiratory syncytial virus (RSV), *n* (%)	117 (5.29)	79 (4.04)	38 (14.67)	
human adenovirus (HAdV), *n* (%)	95 (4.29)	84 (4.30)	11 (4.25)	
Human metapneumovirus (HMPV), *n* (%)	68 (3.07)	67 (3.43)	1 (0.39)	
Human rhinovirus (HRV), *n* (%)	35 (1.58)	20 (1.02)	15 (5.79)	
Human bocavirus (HBoV), *n* (%)	31 (1.40)	29 (1.48)	2 (0.77)	
Human coronavirus (HCoV), *n* (%)	28 (1.27)	25 (1.28)	3 (1.16)	
*Mycoplasma pneumoniae* (MP), *n* (%)	15 (0.68)	10 (0.51)	4 (1.54)	
SARS-CoV-2, *n* (%)	5 (0.23)	0 (0.00)	5 (1.93)	
*Chlamydia pneumoniae* (CP), *n* (%)	0 (0.00)	0 (0.00)	0 (0.00)	
More than one pathogen detected, *n* (%)	54 (2.44)	47 (2.41)	7 (2.70)	

Continuous age was compared using the Mann–Whitney U test; IQR, interquartile range; Statistical significances (*P* < 0.05) are in bold.

**Figure 2:**
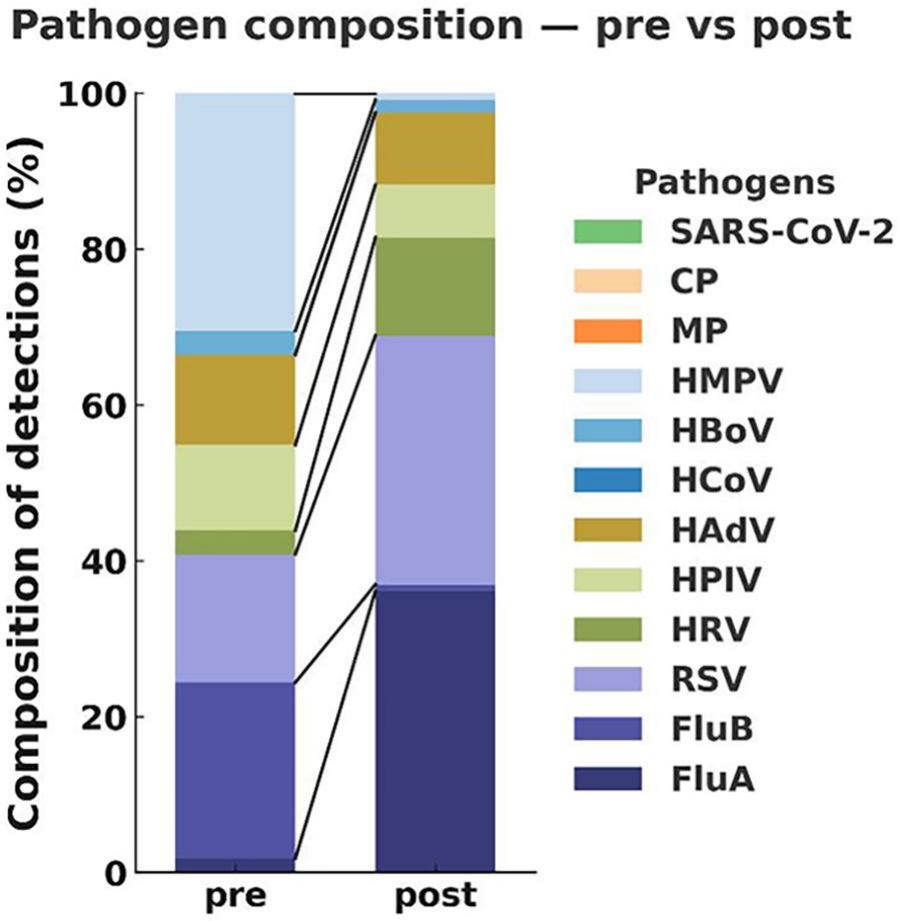
Season-matched comparison of respiratory pathogen detection rates between February–May 2022 and February–May 2023.

To address seasonality, respiratory pathogen detection rates were compared between the same seasonal interval, February–May 2022 and February–May 2023. The overall pathogen detection rate increased significantly from 33.47% (158/472) in 2022 to 47.88% (124/259) in 2023 (*P* < 0.001). FluA, RSV, and HRV showed significantly higher detection rates in 2023 than in 2022. FluA increased from 0.64% in 2022 to 16.60% in 2023, RSV increased from 5.72% to 14.67%, and HRV increased from 1.06% to 5.79% (Table 2). In contrast, FluB decreased from 7.84% to 0.39% and HMPV decreased from 10.59% to 0.39%. These findings indicate a season-matched shift in the detected pathogen spectrum after restrictions were lifted (Figure 2), but the declines in FluB and HMPV may also reflect virus-specific seasonality, regional circulation patterns, and the natural alternation between influenza A and B rather than a direct effect of lifting restrictions alone.

**Table 2:** Season-matched comparison of respiratory pathogen detection rates in children between February–May 2022 and February–May 2023.

Pathogen, *n* (%)	2022 (*n* = 472)	2023 (*n* = 259)	*P*-value
Pathogens detected	158 (33.47)	124 (47.88)	**<0.001**
Influenza A (FluA)	3 (0.64)	43 (16.60)	**<0.001**
Influenza B (FluB)	37 (7.84)	1 (0.39)	**<0.001**
Human parainfluenza virus (HPIV)	18 (3.81)	8 (3.09)	0.609
Respiratory syncytial virus (RSV)	27 (5.72)	38 (14.67)	**<0.001**
Human adenovirus (HAdV)	19 (4.03)	11 (4.25)	0.885
Human metapneumovirus (HMPV)	50 (10.59)	1 (0.39)	**<0.001**
Human rhinovirus (HRV)	5 (1.06)	15 (5.79)	**<0.001**
Human bocavirus (HBoV)	5 (1.06)	2 (0.77)	1.000ᵃ

Human coronavirus (HCoV), *Mycoplasma pneumoniae* (MP), *Chlamydia pneumoniae* (CP), and SARS-CoV-2 had fewer than five detections in both periods; therefore, *P*-values were not calculated. Statistical significances (*P* < 0.05) are in bold.

ᵃ*P*-values were calculated using Fisher's exact test; all others were calculated using Chi-square test.

**Figure 3:**
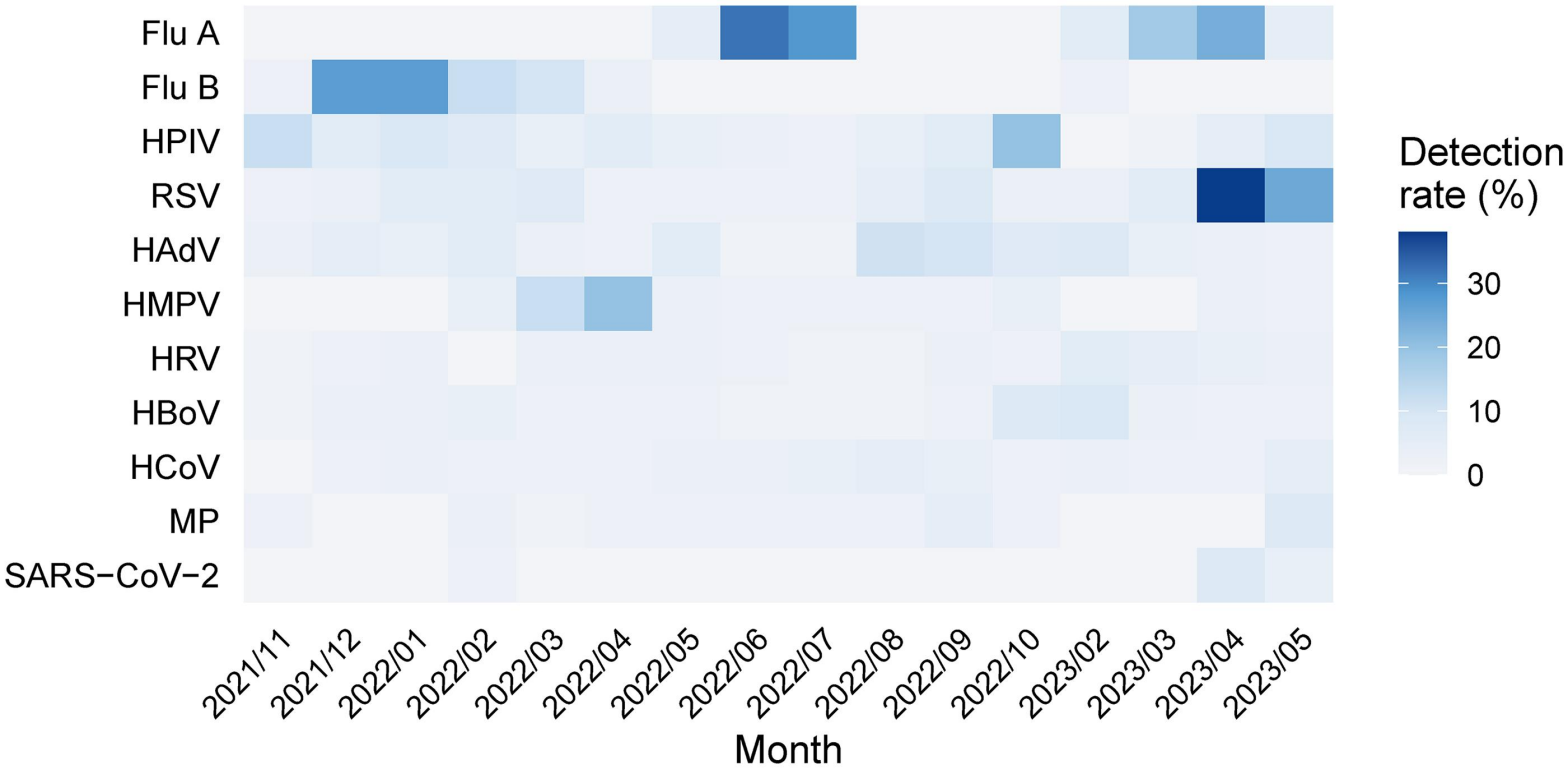
Monthly distribution of respiratory pathogen detection rates in children from February 2022 to May 2023. The figure is intended to show short-term hospital-based trends rather than complete annual seasonality after lifting restrictions.

Monthly detection rates from February 2022 to May 2023 are shown in Figure 3. FluA showed low activity in February–May 2022, a later rise in June–July 2022, and a prominent increase in March–April 2023. RSV and HRV also increased during February–May 2023. In contrast, FluB and HMPV showed limited activity during the 2023 observation window. Because the post-lifting period included only four months, these trends are best interpreted as short-term changes in hospital-based detection patterns rather than complete annual seasonality.

Among pathogens with relatively high detection counts during the season-matched windows (HPIV, RSV, HAdV, and HRV), age distributions were compared between the pre- and post-lifting periods (Table 3). RSV and HPIV cases remained largely concentrated in children younger than 5 years, while HRV was also predominantly detected among the younger age groups. Although the pathogen-specific age distributions did not differ significantly between the two periods, the overall post-lifting tested cohort contained a higher proportion of infants. This age-composition shift is an important potential confounder when interpreting clinical presentation and pathogen detection patterns.

**Table 3:** Season-matched comparison of age distributions for prevalent pathogens among tested children between February–May 2022 and February–May 2023.

Pathogen, *n* (%)	<1 year	1 to <3 years	3 to <5 years	5 to <16 years	Total	*P*-value
Human parainfluenza virus (HPIV)						
Pre-lifting	2 (11.1)	11 (61.1)	2 (11.1)	3 (16.7)	18 (100.0)	0.244
Post-lifting	0 (0.0)	3 (37.5)	4 (50.0)	1 (12.5)	8 (100.0)	
Respiratory syncytial virus (RSV)						
Pre-lifting	4 (14.8)	14 (51.9)	8 (29.6)	1 (3.7)	27 (100.0)	0.921
Post-lifting	6 (15.8)	16 (42.1)	14 (36.8)	2 (5.3)	38 (100.0)	
Human adenovirus (HAdV)						
Pre-lifting	1 (5.3)	5 (26.3)	9 (47.4)	4 (21.1)	19 (100.0)	0.202
Post-lifting	1 (9.1)	7 (63.6)	2 (18.2)	1 (9.1)	11 (100.0)	
Human rhinovirus (HRV)						
Pre-lifting	2 (40.0)	0 (0.0)	3 (60.0)	0 (0.0)	5 (100.0)	0.377
Post-lifting	2 (13.3)	4 (26.7)	6 (40.0)	3 (20.0)	15 (100.0)	

Pre-lifting (02/2022–05/2022); Post-lifting (02/2023–05/2023).

## Discussion

In this retrospective hospital-based study, we used a single-tube multiplex probe amplification assay capable of simultaneous detection of 12 respiratory targets through differential melting curves and fluorescent signatures. The main contribution of this study is the description of a short-term, season-matched shift in pediatric respiratory pathogen detection in subtropical Guangzhou after the lifting of COVID-19 restrictions. A major strength is that the same specimen type, nucleic-acid extraction system, PCR instrument, and pathogen panel were used throughout the study period, reducing the likelihood that technical changes explained the observed differences. Compared with February–May 2022, February–May 2023 was characterized by higher detection of FluA, RSV, and HRV. At the same time, the post-lifting tested population was younger and included more children with wheezing and lower respiratory tract diagnoses, which may reflect both true changes in respiratory infections and changes in health-care-seeking or admission behavior.

Our findings are consistent with recent international and Chinese reports showing that the relaxation of non-pharmaceutical interventions was followed by disrupted seasonality and rebounds of selected pediatric respiratory infections. A multinational European time-series analysis reported increases in pediatric respiratory tract infections after NPI lifting, and large Chinese hospital-based studies reported post-pandemic increases in FluA, RSV, adenovirus, and rhinovirus detections. However, the specific pathogen pattern differed across regions and surveillance systems, emphasizing that local climate, school reopening, population immunity, testing access, and admission thresholds must be considered when interpreting post-pandemic respiratory-virus dynamics (9–15).

The lower detection of FluB and HMPV in February–May 2023 should be interpreted cautiously. Influenza A and B often show alternating predominance and may not strongly co-circulate in the same short season. Similarly, HMPV circulation can vary substantially by region and year. Therefore, the observed decreases in FluB and HMPV are best viewed as part of a broader change in the respiratory-virus ecosystem rather than as direct causal consequences of policy relaxation alone. This interpretation is supported by studies from other settings where FluB or HMPV resurged after prolonged inactivity, highlighting that post-pandemic respiratory-virus dynamics are heterogeneous rather than uniform (16–18).

The higher proportion of infants in the post-lifting cohort is clinically important and has direct implications for pediatric services. Infants may have had fewer previous exposures to common respiratory viruses during periods of reduced social mixing, and their immune systems are still developing. This may increase susceptibility when community transmission resumes. Nevertheless, age composition can confound comparisons of clinical presentation and pathogen positivity. Therefore, the observed increases in lower respiratory tract diagnoses, fever, and wheezing should be interpreted cautiously and not as definitive evidence of increased intrinsic disease severity. Clinically, the findings support maintaining RSV and influenza surveillance, strengthening vaccination and prevention strategies where applicable, and preparing pediatric outpatient and inpatient services for short-term surges among infants and young children.

SARS-CoV-2 circulation is another important contextual factor. The pre-lifting period began while SARS-CoV-2 was already circulating, and SARS-CoV-2 infection itself, as well as associated public-health responses, could have altered the transmission of other respiratory pathogens. In addition, hospital-based SARS-CoV-2 assays were suspended from November 2022 to February 2023, while the major community outbreak occurred from December 2022 to January 2023. Therefore, the low SARS-CoV-2 detection rate in this dataset most likely reflects a timing mismatch and testing-policy changes rather than absence of local SARS-CoV-2 spread (19).

This study has several limitations. First, the full pre-lifting and post-lifting periods were unequal in length, and the post-lifting window covered only February–May 2023; therefore, annual incidence rates could not be estimated. Second, this was a single-center hospital-based study, and changes in health-care-seeking behavior, testing accessibility, and admission thresholds may have influenced the composition of tested children. Third, the post-lifting cohort contained a higher proportion of infants, and individual-level data on vaccination status, prior infection history, prematurity, detailed disease severity scores, and community denominators were unavailable. Fourth, SARS-CoV-2 circulation and changes in SARS-CoV-2 testing policy may have affected both pathogen circulation and ascertainment. Fifth, the analysis was primarily descriptive and used unadjusted comparisons; therefore, it cannot establish causality between policy lifting and pathogen-specific changes. These limitations restrict causal inference, but the season-matched comparison still provides useful evidence of short-term changes in pediatric respiratory pathogen detection after lifting COVID-19 restrictions.

## Conclusion

After the lifting of COVID-19 restrictions, pediatric respiratory pathogen detection in Guangzhou showed a season-matched shift toward higher FluA, RSV, and HRV detection, accompanied by a younger tested population and more lower-respiratory clinical diagnoses. These findings support sustained multiplex pathogen surveillance and age-targeted prevention strategies, particularly for infants and young children. Because of residual confounding by seasonality, SARS-CoV-2 circulation, and health-care-seeking behavior, the findings should be interpreted as hospital-based detection patterns rather than definitive population-level incidence or causality.

## Data Availability

The raw data supporting the conclusions of this article will be made available by the authors, without undue reservation.
